# Gender Biases Toward People With Difficulty in Balancing Work and Family Due to ADHD: Two Case Vignette Randomized Studies Featuring Japanese Laypersons and Psychiatrists

**DOI:** 10.7759/cureus.34243

**Published:** 2023-01-26

**Authors:** Ryuhei So, Misuzu Nakashima, Jane Pei-Chen Chang, Marcus P.J. Tan, Ryoma Kayano, Yasuyuki Okumura, Toru Horinouchi, Toshitaka Ii, Toshihide Kuroki, Tsuyoshi Akiyama

**Affiliations:** 1 Health Promotion and Human Behavior, School of Public Health in the Graduate School of Medicine, Kyoto University, Kyoto, JPN; 2 Graduate School of Human-Environment Studies, Kyushu University, Fukuoka, JPN; 3 Department of Clinical Research, National Hospital Organization Hizen Psychiatric Medical Center, Saga, JPN; 4 Department of Psychiatry, China Medical University Hospital, Taichung, TWN; 5 Institute of Psychiatry, Psychology and Neuroscience, King's College London, London, GBR; 6 Department of Child and Adolescent Psychiatry, Institute of Psychiatry, Psychology and Neuroscience, King's College London, London, GBR; 7 Department of Child and Adolescent Psychiatry, South London and Maudsley NHS Foundation Trust, London, GBR; 8 Center for International Collaborative Research, Nagasaki University, Nagasaki, JPN; 9 Epidemiology, Initiative for Clinical Epidemiological Research, Tokyo, JPN; 10 Department of Psychiatry, Hokkaido University Graduate School of Medicine, Hokkaido, JPN; 11 Department of Psychiatry, Aichi Medical University, Nagoya, JPN; 12 Department of Neuropsychiatry, NTT Medical Center Tokyo, Tokyo, JPN

**Keywords:** adult attention deficit hyperactivity disorder (adhd), labor force participation, gender gap, randomized controlled trial, gender bias

## Abstract

Objective

The gender gap in labor force participation is likely larger in adults with attention deficit hyperactivity disorder (ADHD) than that in the general population. Thus, we investigated whether gender affected the perception toward persons displaying ADHD symptoms and experiencing difficulty in balancing work and family.

Methods

Both Japanese laypersons and psychiatrists were recruited for web-based surveys in March and October 2020 via an online survey company, Cross Marketing Inc., and the secretariat of the Japanese Society of Psychiatry and Neurology, respectively. The participants were randomly assigned to read either a male or female case vignette. The vignettes were identical, except for the gender of the patient in the case. The primary and secondary outcomes were the respondents’ opinions on the seriousness of the case and the degree to which the case’s wish should be maintained, using sliding scales of 0-100.

Results

We included 560 laypersons and 585 psychiatrists. Neither cohort differed in most outcomes between the groups assigned to the male and female case vignettes. Among laypersons, the average score of seriousness was 58.8 in the female-vignette group and 58.6 in the male-vignette group (mean difference, 0.15; 95% confidence interval, -4.9 to 5.2). Among psychiatrists, the average score of seriousness was 53.9 in the female-vignette group and 53.7 in the male-vignette group (mean difference, 0.18; 95% confidence interval, -3.1 to 3.4). Similarly, between-group differences in the opinions on the degree to which the case’s wish should be maintained were 1.2 in laypersons and 0.63 in psychiatrists. We found no significant interaction between the gender of the case and the respondent’s gender in any of the outcomes.

Conclusion

Our results did not support the hypothesis that women were more likely to be pressured to prioritize family over work than men were when there was difficulty balancing work and family due to ADHD symptoms.

## Introduction

The gender gap in the economy remains wide [1]. Globally, the proportion of labor force participation is reportedly only 55% for adult females and 80% for adult males [2]. The gender gap in labor force participation varies significantly between countries, possibly due to differences in social norms regarding gender roles [3].

The gender gap in labor force participation may be larger in adults with attention deficit hyperactivity disorder (ADHD) when compared to the general population. In a clinic-based study in Norway, the risk of unemployment due to ADHD symptoms was twice as high in females than in males [4]. A population-based study in Canada also showed that unemployed females exhibited significantly greater ADHD symptoms than employed females did, although no significant difference in the severity of ADHD symptoms was found for males between full-time workers and unemployed people [5]. A previous study suggested that severe ADHD symptoms are associated with unemployment in females only.

However, it is not clear why females with ADHD face more difficulties in labor force participation than males with the same difficulties. A previous study proposed the hypothesis that females have more difficulties than males in employment because of the social norm that females are expected to prioritize family over work [6].

Thus, we aimed to assess the hypothesis that women are more likely to be pressured to prioritize family over work when there was difficulty balancing work and family due to ADHD symptoms. Consequently, we conducted two case vignette randomized controlled studies with Japanese laypersons and psychiatrists using male and female ADHD case vignettes. We assumed that a larger gender gap may be found in the study with laypersons than in the study with psychiatrists because psychiatrists are trained in shared decision-making in response to patient needs and social contexts.

The preliminary results of these studies were previously presented as an annual meeting abstract at the Japanese Society of Psychiatry and Neurology (JSPN) Annual Meeting on September 19, 2021.

## Materials and methods

Setting and design

Two web-based cross-sectional randomized case vignette studies with Japanese laypersons and psychiatrists were conducted from March 12, 2020, to March 16, 2020, and from October 5, 2020, to October 24, 2020. We did not disclose our hypotheses to the potential participants. The respondents were told that the study aimed to investigate the factors that could influence their impression of a psychiatric disorder. All the survey processes, including informed consent, were completed online. The study protocol was approved by the Institutional Review Board of Kyushu University (approval number: 19015) and registered with the University Hospital Medical Information Network Clinical Trials Registry (UMIN000039690) on March 4, 2020. Similarly, the Institutional Review Board of Okayama Psychiatric Medical Center approved the study protocol with psychiatrists (approval number: 30-27) and registered with the University Hospital Medical Information Network Clinical Trials Registry (UMIN000041931) on September 29, 2020.

Participants and recruitment

Regarding laypersons, the eligibility criteria were adults who lived in Japan and were aged 20 years or older. The respondents were randomly sampled from a survey panel owned by the online survey company Cross Marketing Inc. The survey panel included more than two million respondents who had participated in some surveys within a year. Participants were offered a small cash incentive to complete the survey. Although the authors were not informed of the incentives for the respondents, we estimated that it would be less than a few hundred yen per respondent based on the total cost of the survey and the number of respondents. The process of this study was as follows: (1) Cross Marketing Inc. invited potential participants for the survey panel who met the eligibility criteria of our study; (2) some of the invited potential participants accessed our study website to read the informed consent form; and (3) they expressed their consent to participate in the study by clicking the button on the informed consent page that states “I read the informed consent form and consent to participate in this study of my own free will.” The study website was developed using cloud-based survey software (Survey Monkey®, Momentive Inc., San Mateo, California, USA).

As for the study with psychiatrists, the eligibility criterion was psychiatrists belonging to the JSPN, which is the largest psychiatric academic society in Japan. To recruit respondents, we sent email invitations to all members of the JSPN with support from the secretariat of society. No incentives were offered to the psychiatrists who participated in the study. Similar to the study with laypersons, respondents consented to participate in the study through the website developed using Survey Monkey®.

Randomization, allocation concealment, and blinding

The two studies were conducted using common processes of randomization, allocation concealment, and blinding. Upon clicking on the consent button, the participants were randomly assigned to read either the male or female case vignette in a 1:1 ratio using the Survey Monkey function. Allocation concealment was guaranteed via an automatic random allocation without human involvement.

We did not disclose that our study aimed to investigate whether the gender of the patient affected the assessment of the impairment of social functioning due to undiagnosed ADHD. Instead, we only revealed that this study aimed to investigate the factors that could influence the impression of mental disorders. The principal investigator (RS) was not blinded to the allocations when statistical analyses were performed after data collection.

Case vignettes

The case vignette presented to respondents was either a female or male middle-aged case with trouble at home and work due to undiagnosed ADHD. Except for the description related to the gender of the patient in the case, the two vignettes were identical. Although the situations of the cases presented to laypersons and psychiatrists were almost the same, “his/her behavior during the initial interview” was added to the case vignette for psychiatrists (Appendix).

The development process of the case vignettes was as follows: (1) MN, a psychologist involved in clinical and research work on ADHD for 20 years, drafted the case vignettes in Japanese under the supervision of TK, a psychiatrist, and professor of clinical psychology practice with more than 30 years of clinical experience; (2) all the researchers reviewed and commented on the case vignettes translated into English by RK; and (3) MN revised both the English and Japanese versions of the case vignettes according to the comments. This process was repeated until all researchers agreed on the contents of the case vignettes between the male and female versions. In this study, only Japanese vignettes were distributed to participants.

Outcomes

The primary and secondary outcomes were the respondents’ opinions on the seriousness of the case’s problem and the degree to which the case’s wish should be maintained, using sliding scales from 0 to 100. To measure the primary and secondary outcomes, we asked laypersons the following: “If you were his/her friend and he/she came to you for advice, (1) to what extent should he/she consult a psychiatrist in your opinion on a sliding scale of 0 to 100 (0: nothing necessary, 100: extremely necessary)? (Please answer not what you advise to him, but what you think by placing the necessity of his/her psychiatric consultation on a scale of 0 to 100. 0: nothing necessary, 100: extremely necessary); (2) When you advise him/her, to what degree will you act on his/her intention to maintain his/her wish to not have his/her work hours reduced? (Please answer to what degree you will adopt the patient’s wishes on a scale of 0 to 100. 0: encourage him/her to rethink his/her wishes, 100: adopt his/her wishes completely).” Both outcomes were measured using a sliding scale (0-100).

Regarding the study with psychiatrists, we asked the following questions to assess the primary and secondary outcomes: “From your experience seeing these kinds of patients, how seriously do you think her mental condition is at this time? (Please answer by placing the seriousness of her condition on a scale of 0 to 100. 0: nothing problematic, 100: extremely problematic); (2) When you make a mutual understanding of the treatment goals of this patient, to what degree will you act on her intention of maintaining her wish not to have her work hours reduced? (Please answer to what degree you will adopt the patient’s wishes on a scale of 0 to 100. 0: encourage her to rethink her wishes, 100: adopt her wishes completely)”. Both outcomes were measured using a sliding scale (0-100).

Variables

We collected the same characteristic information of the respondents in the two studies: gender, age, having a partner or not, partner's working status, and the number of children. When respondents submitted the form, the survey system automatically checked that all the required fields had been filled.

Sample size

We assumed that the between-group standardized mean difference in the primary outcome was 0.25 for both studies with laypersons and psychiatrists. Thus, the target sample sizes for both studies were 252 per group and 504 in total, with a statistical power of 80% and a two-sided significance level of 5%.

Statistical analysis

**Figure 1:**
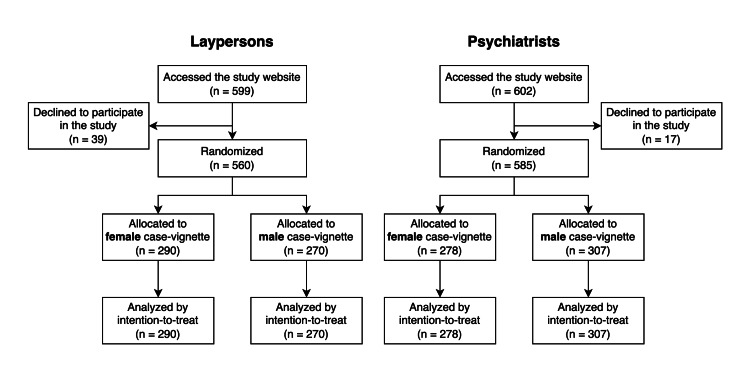
Participant flow

We described a Consolidated Standards of Reporting Trials (CONSORT) flowchart (Figure 1), which included the number of people who accessed the study websites and were randomized and analyzed. We calculated descriptive statistics of the respondents’ characteristics for each study.

For the two studies, we drew violin plots to show the distribution of scores of the primary and secondary outcomes. We then calculated the between-group standardized mean differences and 95% confidence intervals (CI) for these outcomes. For significance testing, we conducted a student’s t-test for both outcomes.

To investigate the hypothesis that the gender of the respondents affected their opinion of the case vignette, we planned a subgroup analysis by gender for both the primary and secondary outcomes. Our expectation was that male respondents would rely more on social norms about gender roles than female respondents. Thus, gender would have a greater influence on male respondents when compared to female respondents. This subgroup analysis was only going to be conducted if there was a statistically significant interaction in the multiple regression findings between the gender of the case vignette and that of the respondents. To test the significance of the interaction, we set the alpha level to 0.20.

## Results

We included 560 respondents in the study with laypersons and 585 respondents in the study with psychiatrists, as shown in the CONSORT flowchart (Figure 1). Table 1 summarizes the characteristics of the respondents included in the two studies whose data were analyzed.

**Table 1:** Respondents’ characteristics

	General Population		Psychiatrists	
Characteristic	Female case (N = 290)	Male case (N = 270)	Female case (N = 278)	Male case (N = 307)
Respondent’s gender				
Female, n (%)	152 (52%)	133 (49%)	73 (26%)	66 (21%)
Age, years, n (%)				
20–24	40 (14%)	47 (17%)	NA	NA
25–34	45 (15%)	39 (14%)	42 (15%)	42 (14%)
35–44	53 (18%)	43 (16%)	85 (31%)	86 (28%)
45–54	51 (18%)	52 (19%)	82 (29%)	90 (29%)
55–64	41 (14%)	28 (10%)	45 (16%)	65 (21%)
65–74	22 (7.6%)	33 (12%)	22 (7.9%)	18 (5.9%)
≥ 75	39 (13%)	31 (11%)	2 (0.7%)	6 (2.0%)
Has a partner, n (%)	141 (48%)	134 (49%)	230 (83%)	256 (83%)
Partner’s working status, n (%)				
Working	92 (65%)	85 (63%)	161 (58%)	160 (52%)
Has children, n (%)	129 (44%)	131 (48%)	190 (68%)	216 (70%)

**Figure 2:**
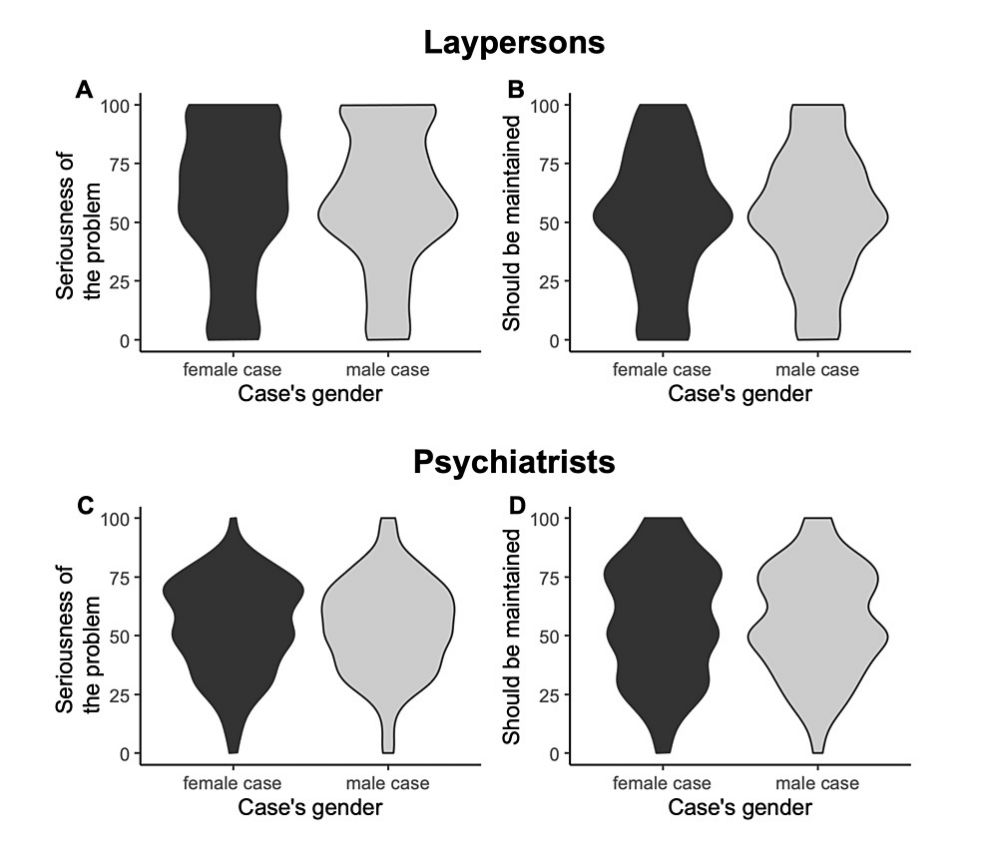
Distributions of the scores for the primary and secondary outcomes The y-axis of A represents the answer to the question, “To what extent should he/she consult a psychiatrist in your opinion on a sliding scale of 0 to 100 (0: nothing necessary, 100: extremely necessary)? (Please answer not what you advise to him but what you think by placing the necessity of his/her psychiatric consultation on a scale of 0 to 100. 0: nothing necessary, 100: extremely necessary)” The y-axis of B represents the answer to the question, “When you advise him/her, to what degree will you act on his/her intention to maintain his/her wish to not have his/her work hours reduced? (Please answer the degree to which you will adopt the patient’s wishes on a scale of 0 to 100. 0: encourage him/her to rethink his/her wishes; 100: adopt his/her wishes completely).” The y-axis of C represents the answer to the question, “From your experience seeing these kinds of patients, how seriously do you think her mental condition is at this time? (Please answer by placing the seriousness of her condition on a scale of 0 to 100. 0: not problematic, 100: extremely problematic)” The y-axis of D represents the answer to the question, “When you make a mutual understanding of the treatment goals with this patient, to what degree will you act on her intention to maintain her wish to not have her work hours reduced? (Please answer the degree to which you will adopt the patient’s wishes on a scale of 0 to 100. 0: encourage her to rethink her wishes; 100: adopt her wishes completely).”

The primary and secondary outcomes did not significantly differ between groups in the two studies (Table 2 and Figure 2). In the study with laypersons, the average score of seriousness, which was the primary outcome, was 58.8 in the male-vignette group and 58.6 in the female-vignette group (mean difference, 0.15; 95% CI, -4.9 to 5.2). In the study with psychiatrists, the average score of seriousness was 53.9 in the female-vignette group and 53.7 in the male-vignette group (mean difference, 0.18; 95% CI, -3.1 to 3.4). As for the secondary outcome, the between-group mean differences in the opinions on the degree to which the case’s wish should be maintained were -1.2 (95% CI, -5.8 to 3.5) and 0.63 (95% CI, -3.2 to 4.5) in the study with laypersons and psychiatrists, respectively. Standardized mean differences in the primary and secondary outcomes of the two studies are shown in Table 2.

**Table 2:** Main analyses on the primary and secondary outcomes ^1^The answer to the question “To what extent should he/she consult a psychiatrist in your opinion, on a sliding scale of 0 to 100 (0: nothing necessary, 100: extremely necessary)? (Please answer not what you advise to him but what you think by placing the necessity of his/her psychiatric consultation on the scale of 0 to 100. 0: nothing necessary, 100: extremely necessary).” ^2^The answer to the question “When you advise him/her, to what degree will you act on his/her intention of maintaining his/her wish to not have his/her work hours reduced? (Please answer to what degree you will adopt the patient’s wishes on the scale of 0 to 100. 0: encourage him/her to rethink his/her wishes, 100: adopt his/her wishes completely).” ^3^The answer to the question “From your experience seeing these kinds of patients, how serious do you think her mental condition is at this time? (Please answer by placing the seriousness of her condition on the scale of 0 to 100. 0: nothing problematic, 100: extremely problematic).” ^4^The answer to the question “When you make a mutual understanding on the treatment goals with this patient, to what degree will you act on her intention of maintaining her wish to not have her work hours reduced? (Please answer to what degree you will adopt the patient’s wishes on the scale of 0 to 100. 0: encourage her to rethink her wishes, 100: adopt her wishes completely).” N: number; SD: standardized deviation; MD: mean difference; SMD: standardized mean difference; CI: confidence interval

Layperson				
	Female case (N = 290) Mean (SD)	Male case (N = 270) Mean (SD)	MD (95% CI)	SMD (95% CI)
Seriousness of the problem (0−100)^1^	58.8 (31.1)	58.6 (29.6)	0.15 (-4.9 to 5.2)	0.01 (-0.16 to 0.17)
His/Her wish should be maintained (0−100)^2^	51.9 (28.3)	53.1 (27.4)	-1.2 (-5.8 to 3.5)	-0.04 (-0.21 to 0.12)
Psychiatrist				
	Female case (N = 278) Mean (SD)	Male case (N = 307) Mean (SD)	MD (95% CI)	SMD (95% CI)
Seriousness of the problem (0-100)^3^	53.9 (20.0)	53.7 (20.0)	0.18 (-3.1 to 3.4)	0.01 (-0.15 to 0.17)
His/Her wish should be maintained (0-100)^4^	56.0 (24.8)	55.3 (22.6)	0.63 (-3.2 to 4.5)	0.03 (-0.14 to 0.19)

The results of the ad hoc interaction analyses in the study with laypersons and psychiatrists are summarized in Table 3. No significant interaction effect was demonstrated with regard to the primary or secondary outcomes in either study. Thus, the planned subgroup analyses by respondents’ gender were not performed according to predetermined conditions.

**Table 3:** Interaction between the case’s gender and respondent’s characteristics ^1^Each model contained the case’s gender, each characteristic, and an interaction term of the case’s gender and each characteristic as independent variables. ^2^The answer to the question “To what extent should he/she consult a psychiatrist in your opinion, on a sliding scale of 0 to 100 (0: nothing necessary, 100: extremely necessary)? (Please answer not what you advise to him but what you think by placing the necessity of his/her psychiatric consultation on the scale of 0 to 100. 0: nothing necessary, 100: extremely necessary).” ^3^The answer to the question “When you advise him/her, to what degree will you act on his/her intention of maintaining his/her wish to not have his/her work hours reduced? (Please answer to what degree you will adopt the patient’s wishes on the scale of 0 to 100. 0: encourage him/her to rethink his/her wishes, 100: adopt his/her wishes completely).” N: number; SD: standardized deviation; SMD: standardized mean difference; CI: confidence interval

	Seriousness of the problem^2^			His/Her wish should be maintained^3^		
Characteristic^1^	Beta	95% CI	p-value	Beta	95% CI	p-value
Male case * male respondent in the study with laypersons	-1.9	-12.0 to 8.2	0.71	-3.6	-13.0 to 5.6	0.44
Male case * male respondent in the study with psychiatrists	0.55	-7.1 to 8.2	0.9	2.0	-7.1 to 11	0.7

## Discussion

In both studies with laypersons and psychiatrists, there were no differences in most of the outcomes between respondents assigned to the male case vignette and female case vignette. These results suggest that literal gender differences do not influence the opinions of Japanese laypersons and psychiatrists.

There are some possible explanations for these null results. First, even in the general population of Japan, where the gender gap is large, people may no longer behave according to the old-fashioned social norm that females should prioritize housework and childcare over work outside their homes from a third person or outsider’s point of view [1,7]. Second, we asked the respondents to provide their opinions on the case vignette as a friend of the patient in the case. In other words, respondents did not have to consider being personally involved in the problems caused by the case. This might have resulted in a more generous attitude, especially in comparison to if they were to answer from the standpoint of someone with more interest or stake, such as a spouse or colleague in the workplace. When a case’s gender has little influence on the opinions of laypersons, the null results of the study with psychiatrists are less surprising because they are trained to separate attitudes as professionals from their personal views. The third possible explanation is that we failed to capture the gender bias among respondents that actually existed. The written description of our case vignette may not have sufficiently evoked the image of the gender of the case to the respondents, although previous studies have revealed a significant effect of the case’s gender on respondents using written case vignettes [8,9].

These two studies had several limitations. The first limitation is the generalizability of our results because our study only included Japanese respondents. However, the null results from these studies in Japan, whose gender gap index ranked 120th out of 156 countries, may be applicable to other countries with a lower gender gap [1]. Second, we may have failed to capture gender bias within the respondents because of the lack of reality in the written case vignette. The results could have been different if a more vivid case vignette with pictures or videos had been used. Third, we investigated only one aspect of gender bias that could affect the labor participation of people with ADHD. The respondents were asked to answer questions as a third person or outsider, thus, gender bias in people with ADHD and their significant others has yet to be examined in future studies.

## Conclusions

In conclusion, the present randomized case vignette studies did not support the hypothesis that females are more likely to be pressured to prioritize family over work than males are when it was difficult to balance work and family due to ADHD symptoms. Further studies are needed to explore other ways in which gender disparity may manifest in the labor participation of people with ADHD.

## Appendices

Case vignette presented to laypersons

Please read the following case from the standing point of his/her friend.

Case: “Mr./Ms. A, 34 years old male office worker, who lives with his/her wife/husband and a 5-year-old child.”

Issue: Troubles at work and home

The situation in his/her workplace

After graduating from his/her university he/she has been working as an administrative staff at a school. Due to his/her poor management and planning skills, he/she has not been promoted. Despite working many days overtime, he/she often misses deadlines, makes mistakes, and loses documents. He/She is friendly with colleagues in other departments, but due to the fact that the co-workers in his/her department always have to correct his/her mistakes, the social relation at work suffers.

Personal history

His/Her poor planning skills have been observed since he/she started attending school. In his/her school report, general carelessness (i.e., leaving things, lack of concentration) is indicated by multiple homeroom teachers. Despite these problems, he/she got good grades.

He/She has always had trouble maintaining an orderly living space; his/her parents helped him clean his/her room until he/she married at 29. Although he/she didn’t collect or hoard things, his/her room was always messy with clothes and textbooks scattered on the floor.

He/She was able to graduate from university in 4 years by having friends who helped him with courses and other procedures. After graduation, he/she started working for a school. His/Her mistakes were overlooked due to his/her s being a new employee. After 7 years (when he/she married) with no improvement at his/her workplace, the situation worsened; this coincided with troubles at home.

Situation at home

Although his/her wife/husband shares the housework, he/she is still unable to keep up his/her end. He/She misses deadlines for submitting school documents for his/her child or loses those school documents. His/Her wife/husband says “Because you are always working overtime, you are unable to help me with the housework, and I can’t do all of it alone because I’m also tired from working. Since you are not able to help with the housework due to the overtime at your job, you should consider how to become a better employee.” They often fight about this. However, he/she denied experiencing any psychological or physical abuse within his/her home environment.

Although he/she has always made mistakes at work, the situation got worse after marriage. His/Her supervisor repeatedly has instructed him to improve his/her organizational skills. He/She would apologize and hope to improve but the situation did not improve, thus continuing to miss deadlines and creating problems for his/her co-workers.

From this history, his/her supervisor recommended him to see a psychiatrist suspecting that he/she had long-standing psychological issues.

If you were his/her friend and he/she came to you for advice,

1. To what extent should he/she consult a psychiatrist in your opinion, on a sliding scale of 0 to 100 (0: nothing necessary, 100: extremely necessary)? (please answer not what you advise to him but what you think by placing the necessity of his/her psychiatric consultation on a scale of 0 to 100. 0: nothing necessary, 100: extremely necessary)

0------------------------------------------------------------------------100

2. When you advise him/her, to what degree will you act on his/her intention of maintaining his/her wish not to have his/her work hours reduced? (please answer to what degree you will adopt the patient’s wishes on a scale of 0 to 100. 0: encourage him/her to rethink his/her wishes, 100: adopt his/her wishes completely.)

0------------------------------------------------------------------------100

Case vignette presented to psychiatrists

Please read the following case from the standing point of his/her friend.

Case: “Mr./Ms. A, 34 years old male office worker, who lives with his/her wife/husband and a 5-year-old child.”

Chief complaint: Troubles at work and home

The situation in his/her workplace

After graduating from his/her university he/she has been working as an administrative staff at a school. Due to his/her poor management and planning skills, he/she has not been promoted. Despite working many days overtime, he/she often misses deadlines, makes mistakes, and loses documents. He/She is friendly with colleagues in other departments, but due to the fact that the co-workers in his/her department always have to correct his/her mistakes, the social relation at work suffers.

Personal history

His/Her poor planning skills have been observed since he/she started attending school. In his/her school report, general carelessness (i.e. leaving things, lack of concentration) is indicated by multiple homeroom teachers. Despite these problems, he/she got good grades.

He/She has always had trouble maintaining an orderly living space; his/her parents helped him clean his/her room until he/she married at 29. Although he/she didn’t collect or hoard things, his/her room was always messy with clothes and textbooks scattered on the floor.

He/She was able to graduate from university in 4 years by having friends who helped him with courses and other procedures. After graduation, he/she started working for a school. His/Her mistakes were overlooked due to his/her s being a new employee. After 7 years (when he/she married) with no improvement at his/her workplace, the situation worsened; this coincided with troubles at home.

Situation at home

Although his/her wife/husband shares the housework, he/she is still unable to keep up his/her end. He/She misses deadlines for submitting school documents for his/her child or loses those school documents. His/Her wife/husband says “Because you are always working overtime, you are unable to help me with the housework, and I can’t do all of it alone because I’m also tired from working. Since you are not able to help with the housework due to the overtime at your job, you should consider how to become a better employee.” They often fight about this. However, he/she denied experiencing any psychological or physical abuse within his/her home environment.

Although he/she has always made mistakes at work, the situation got worse after marriage. His/Her supervisor repeatedly has instructed him to improve his/her organizational skills. He/She would apologize and hope to improve but the situation did not improve, thus continuing to miss deadlines and creating problems for his/her co-workers.

Visiting psychiatrist

From this history, his/her supervisor recommended him to see a psychiatrist suspecting that he/she had long-standing psychological issues. She was also becoming aware of her unusual character, so she decided to visit a psychiatric clinic alone.

Her behavior during the initial interview

She tended to talk too much, often going off on tangents, but she could respond to your questions directly but briefly; she was able to describe her recent situation while maintaining eye contact and using non-verbal communication appropriately. “I want to continue my work and I do not want my hours reduced because I like it.” She tells you how she enjoys her work and wants to continue in her current job. She also says she does not want to reduce her current workload or work on a reduced timetable. There were no unusual disturbances in mood, psychotic symptoms, extreme anxiety, panic attack, or problems with her communication

There were no concerns with her physical health.

If you were the doctor in charge of her,

1. From your experience seeing these kinds of patients, how seriously do you think her mental condition is at this time? (please answer by placing the seriousness of her condition on a scale of 0 to 100. 0: nothing problematic, 100: extremely problematic)

0------------------------------------------------------------------------100

2. When you make a mutual understanding of the treatment goals with this patient, to what degree will you act on her intention of maintaining her wish not to have her work hours reduced? (Please answer to what degree you will adopt the patient’s wishes on a scale of 0 to 100. 0: encourage her to rethink her wishes, 100: adopt her wishes completely.)

0------------------------------------------------------------------------100
